# Natural History of Pediatric Idiopathic Histaminergic Angioedema: A Retrospective Monocentric Study

**DOI:** 10.3390/children12050600

**Published:** 2025-05-04

**Authors:** Vanessa Migliarino, Alessandro Zago, Camilla Martelossi, Egidio Barbi, Manuela Giangreco, Irene Berti, Laura Badina

**Affiliations:** 1 Azienda Sanitaria Universitaria Friuli Centrale, San Daniele, 33100 Udine, Italy; v.migliarino@libero.it; 2 Department of Medicine, Surgery and Health Sciences, University of Trieste, 34137 Trieste, Italy; egidio.barbi@burlo.trieste.it; 3 Department of Internal Medicine and Medical Specialities—DIMI, University of Genoa, 16132 Genoa, Italy; 5733235@studenti.unige.it; 4 Institute for Maternal and Child Health-IRCCS “Burlo Garofolo”, 34137 Trieste, Italy; manuela.giangreco@burlo.trieste.it (M.G.); irene.berti@burlo.trieste.it (I.B.); laura.badina@burlo.trieste.it (L.B.)

**Keywords:** angioedema, idiopathic histaminergic acquired angioedema, recurrent angioedema, allergy

## Abstract

Background: Idiopathic histaminergic angioedema (IH-AAE) is a pathological entity poorly described in the literature. It overlaps with some forms of chronic urticaria, especially in pediatrics. Objective: This study is a descriptive analysis of this form of angioedema’s natural history and prognosis. The aim is to describe long-term data about the course of this clinical entity, including clinical presentation, recurrence, and response to therapy, emphasizing follow-up and outcome. Methods: We performed a retrospective monocentric descriptive study at the Allergy Unit, Department of Pediatrics of the Institute for Maternal and Child Health of Trieste, Italy. We selected pediatric patients (0–18 years old) visiting the outpatient clinic from January 2010 to December 2020 who received a diagnosis of IH-AAE. We analyzed the disease recurrence, the remission rate, the time and frequency of recurrences, and the body sites involved. Results: The median follow-up was 57 months. Among the 36 individuals examined at follow-up, 9 (25%) still had episodes of angioedema, while 27 (75%) reported the absence of attacks. Disease remission was established in 24 patients (66.6%). The median remission time was 13 months (IQR: 7–28). When comparing AE recurrence at onset and follow-up, in all children, the number of episodes decreased (in 4/9 patients) or remained unchanged over time (in 5/9 patients). Moreover, within this group, AE recurrence was recorded as high, intermediate, and low, respectively, in one (11.1%), two (22.2%), and six patients (66.7%). The median number of monthly episodes was one (IQR: 0.2–3), and eight was the maximum value. The initial recurrence of AE attacks has no impact on the time and rate of remission (*p* = 0.56). According to these data, 36% of the patients will go into remission in 1 year, 54% in 2 years, and 71% in 6.5 years, while 14% of the children will still present with AE after 8 years of disease. Conclusions: IH-AAE is a benign and self-limiting condition that can sometimes last several years. Over time, the number of episodes per month decreases or, at most, remains unchanged. No patients reported disease worsening. The frequency of attacks at onset does not correlate with the possibility of recovery or the remission time.

## 1. Introduction

Recurrent angioedema (AE) may represent a challenging issue for clinicians. It is defined as a self-limiting swelling of deep skin layers, such as subcutaneous or submucosal tissues [1]. It is induced by pro-phlogistic mediators like bradykinin or histamine, or drugs that modify vascular endothelial permeability causing extravasation through the cadherin junctions [2]. Over the years, different forms of angioedema have been discovered [3,4,5]. Different classifications have been considered, identifying the release of bradykinin as the final common pathway of angioedema, secondary to the alteration of the kallikrein–kinin system, such as a congenital or acquired (such as autoimmune and lymphoproliferative disorders) defect of the levels (type I) or function (type II) of gene encoding human C1 esterase inhibitor (SERPING), and mutations in genes encoding factor XII [6], plasmin (HAE-PLG), and kininogen-1 (HAE-KNG). In 2024, a new category of angioedema, related to innate vascular endothelium dysfunction, was introduced. This category includes angioedema due to the mutation of angiopoietin-1 (HAE-ANGPT1), myoferlin (HAE-MYOF), and heparan sulfate-glucosamine 3-O sulfotransfersase (HAE-HS3ST6), which are structural endothelial proteins that bind kininogen. Other types of angioedema are the drug-induced form (secondary to AE inhibitors, NSAIDS, angiotensin receptor–neprilysin peptidase, and tissue plasminogen activators) and AE of unknown mechanism [4].

The previously identified idiopathic histaminergic angioedema (IH-AAE) was considered secondary to histamine production, released by an undisclosed mechanism, and a response to antihistaminic treatment can be detected. In this form, a phenotypical overlap with chronic spontaneous urticaria is reported in a variable percentage of cases [7,8,9]. The most up-to-date classification is based on a cell, rather than a mediator, and considers this form of angioedema to be “mast-cell mediated angioedema”, as histamine is not the only mediator produced by mast cells. There are some cases not responsive to a high-dose antihistamine regimen, which are improved by therapy with omalizumab [4]. The understanding of the mechanism is crucial for disease management, as the different types of bradykinin-mediated angioedema do not respond to treatment with antihistamines or steroids.

This study focused on idiopathic histaminergic acquired angioedema (IH-AAE), which is poorly described in the literature regarding its natural history, especially in children [10,11]. We aimed to portray a descriptive analysis of the long-term clinical manifestations and course of this condition in children, to provide a first response to children and their families about the course of this disease over time, and to provide the pediatrician with new insights into this clinical entity.

## 2. Materials and Methods

We performed a retrospective monocentric descriptive study at the Allergy Unit, Department of Pediatrics of the Institute for Maternal and Child Health of Trieste, Italy. We selected pediatric patients (0–18 years old) visiting the outpatient clinic from January 2010 to December 2020 who had received a diagnosis of IH-AAE. We considered isolated AE principally; 33.3% of the patients reported associated urticaria. Isolated urticaria without AE during attacks, single episodes of angioedema and hereditary, allergic, drug-induced, and infection-induced AE were the exclusion criteria.

Medical reports from the clinical software were evaluated to obtain the initial data, and subsequently, all patients were investigated for their current disease status.

The endpoints of this study were to analyze the clinical course of IH-AAE, to estimate remission rate and time, if predictable according to AE severity at disease onset, and to evaluate whether AE recurrence increased or decreased over time.

The reassessment was performed in May 2021 using a phone call questionnaire to evaluate the evolution or remission of the AE, the median time of remission, and the disease’s intensification over time in terms of AE location and frequency.

### 2.1. Definitions

Idiopathic histaminergic angioedema was described as recurrent angioedema with or without associated wheals not secondary to allergies, trauma, infections, autoimmune conditions, drugs (ACE inhibitor use was excluded through anamnesis), systemic diseases, alterations in the levels or function of C1q inhibitor or FXII mutations, and responsive to anti-histaminergic treatment.

Disease recurrence was defined depending on the number of angioedema episodes per month. Specifically, we arbitrarily classified low-recurrence angioedema as 1 or less than 1 episode per month, intermediate recurrence as more than 1 and less than 4 episodes/month, and high recurrence for 4 or more episodes per month.

Disease remission was defined as the absence of AE for at least 1 year from the last episode if taking an antihistamine when needed or not taking it, or for at least 1 year after stopping regular antihistamine therapy.

### 2.2. Statistical Analysis

Categorical variables were described by reporting frequencies and percentages, while medians and interquartile ranges were used for continuous variables. The Shapiro–Wilk test was applied to evaluate normality in the distribution of continuous variables. Given the small sample size, the association between the outcome of interest and categorical variables was assessed using the Fisher exact test, while to verify whether the distribution of remission time was different between the categories of qualitative variables, the Wilcoxon Mann–Whitney test or the Kruskal–Wallis test were used, depending on the number of modalities.

To evaluate the remission rate from angioedema, the Kaplan–Meier curve was constructed considering the remission time as the time between the onset and the last episode of angioedema or the last fixed antihistamine administration for those who had recovered and the time between onset and the call by healthcare personnel for those who had not recovered. The log-rank test was performed to compare the survival distributions of the angioedema recurrence groups. A *p*-value < 0.05 was considered statistically significant. Statistical analyses were conducted using SAS software, Version 9.4 (SAS Institute Inc., Cary, NC, USA).

### 2.3. Ethics

Approval for data collection was obtained by the Ethical Committee of IRCCS Burlo Garofolo (ECC 49/2022, seduta IRB 02/2022, D.D. 16 March 2022).

## 3. Results

### 3.1. Demographics

A total of 58 patients were eligible for the study, and 22 (38%) were lost at follow-up. The final population examined consisted of 36 patients (62%); Table 1 summarizes the demographic and clinical characteristics at disease onset. The median follow-up was 57 months (IQR).

### 3.2. Disease Course and Remission

Among the 36 individuals examined at follow-up, 9 (25%) still had episodes of angioedema, while 27 (75%) reported the absence of attacks. According to the study definition, disease remission was established in 24 patients (66.6%). The remaining 12 (33.3%) were considered as not having healed.

**Figure 1:**
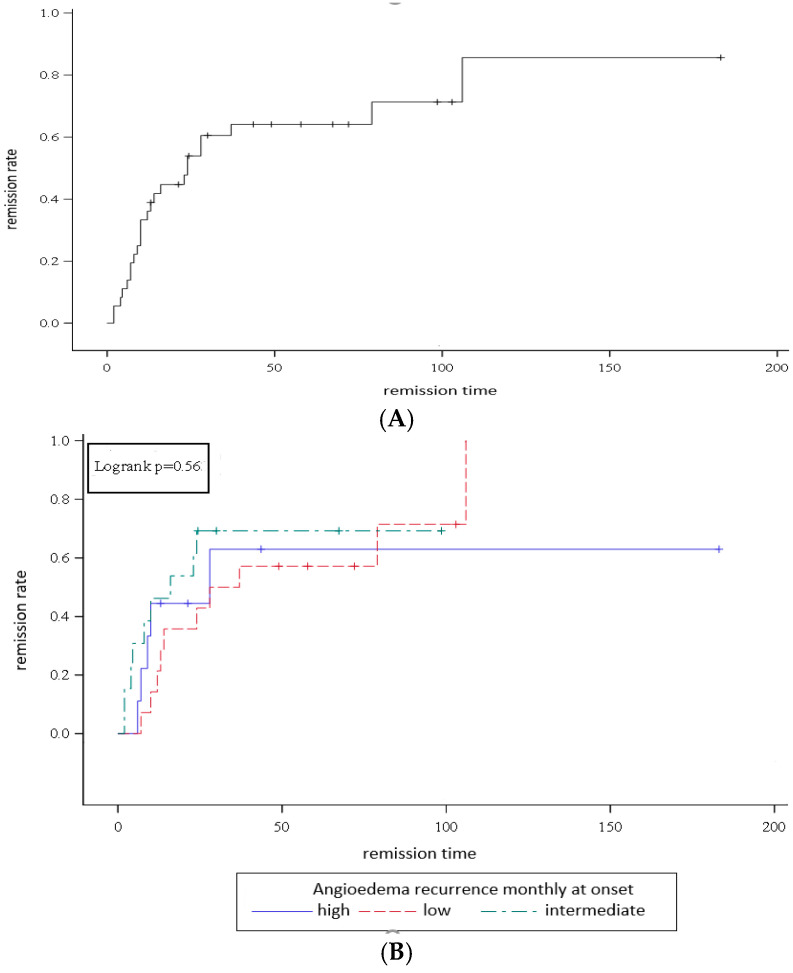
(**A**) Kaplan–Meier curve to estimate remission rate over time (remission time in months). (**B**) Three survival curves for three categories of high-, intermediate-, and low-recurrence AE.

Disease duration varied widely—the median remission time was 13 months (IQR: 7–28), with a duration of at least 2 months and a maximum of 106 months (Figure 1A).

### 3.3. Factors Associated with Disease Remission

When comparing the two groups of healed and unhealed patients, no statistically significant differences were detected between disease remission and the presence of urticaria (*p* = 0.71) or the recurrence of AE episodes per month (*p* = 0.74). Likewise, no statistically significant difference in remission time was revealed, either in high-, intermediate-, low-recurrence (*p* = 0.13) or associated urticaria (*p* = 0.63).

No patient presenting AE reported an intensification of the episodes in frequency or body sites (Table 1). Specifically, when comparing AE recurrence at onset and follow-up, in all children the number of episodes decreased (4/9 patients) or remained unchanged over time (5/9). Moreover, within this group, AE recurrence was recorded as high, intermediate and low, respectively, in one (11.1%), two (22.2%), and six patients (66.7%) (Figure 1B). The median number of monthly episodes was one (IQR: 0.2–3), and eight was the maximum value.

### 3.4. Estimated Remission Rate

The remission rate was defined as the percentage of healed children over the period between disease onset and the last AE episode or the last antihistamine use for healed patients, and over the period between disease onset and the date of the phone call for patients who had not healed, firstly in the whole cohort examined at follow up and then in three distinct subgroups based on AE recurrence at onset. By comparing three survival curves in three categories of high-, intermediate-, and low-recurrence AE (log-rank test), it was possible to state that the initial recurrence of AE attacks has no impact on the time and rate of remission (*p* = 0.56).

## 4. Discussion

Limited data about the long-term outcome of AE in children are available in the literature. With a 57-month median follow-up, this study shows a substantially positive outcome in many patients in a few years. To the best of our knowledge, the only study on AE without urticaria exclusively in children was published by Karagol et al. [12], who investigated this entity with a median follow-up of 17 months among 95 patients. In their study, 51% of the described cases were idiopathic—no one had a life-threatening episode, and only one-third required antihistamine prophylaxis, but all were responsive to antihistamine treatment. Another case series, which included 31 adult patients with a mean age of 50, described the clinical and epidemiological characteristics of idiopathic histaminergic angioedema [13]. The demographic data of our population are similar to the retrospective study by Oak et al. [14], which compares the characteristics of 57 children affected by HAE (hereditary angioedema) with 42 children affected by HA (histaminergic angioedema); more specifically, our study describes a slightly higher age at disease onset (9 years old vs. 7.8 years old). In line with the literature, both studies identify the lips and eyelids as the most common sites involved in this type of angioedema. As expected, abdominal pain and a gastroenterological presentation (36.6% in their study, with percentages reaching 90% in the literature) and extremity involvement are most common in hereditary AE.

IH-AAE has no identifiable cause, or rather the cause cannot be defined as directly linked to mast-cell degranulation and basophil activation (nor the bradykinin-mediated pathway), but has a clear histaminergic etiology. Indeed, the cornerstone of diagnosing IH-AAE is the response to antihistamine treatment. In our study, all of the patients who received antihistamine treatment with different therapeutic schemes showed improvement in terms of their AE episodes. This was an exclusion diagnosis, as patients usually had a silent family and pharmacological history, no association with other medical conditions such as lymphoproliferative disorders, as in the case of acquired AE, and no typical age of onset [15,16,17,18]. Unlike in bradykinin-mediated angioedema, trauma was not a potential trigger of AE episodes.

The condition’s clinical characteristics were highlighted in a Consensus Conference in 2014 [8]. Patients presented recurrent episodes of AE, which developed quickly, reaching their apex six hours after onset, and tending to resolve in around 24 h. Its course over time was usually extrapolated from chronic spontaneous urticaria or reported as expert opinion/experience, assuming it went forward to resolution in 3–5 years from onset in most cases. The rapid onset and resolution of edema allowed us to clinically distinguish this form from the bradykinin-mediated forms, which had instead a slow onset and could persist for several days. The respiratory and gastrointestinal tract, particularly the larynx, were spared as a rule, and no fatal cases were reported. In particular, Faisant’s study and our paper identified the periorbital region and the lips as the most frequently involved sites.

According to previous studies, the long-term outcome was good, as most patients presented a low-intermediate recurrence [19]. Moreover, our results highlighted that the presence of urticaria was not concomitant with the episodes of angioedema, which constituted a diagnostic element of angioedema [7] and a typical characteristic element suggestive of idiopathic histaminergic angioedema [19].

In our study, we highlighted remission in most of the patients within two years. In particular, 36% of the patients went into remission within one year, 54% within two years, and 71% within 6.5 years, while 14% of the children still present AE 8 years after disease onset. However, we could not identify predictors of the natural course nor of remission.

The major limit of this study is the small sample size, which makes it difficult to identify statistically significant associations from the collected data and to generalize the results to a larger population. It could be argued that only 24 patients have isolated angioedema, while the others have angioedema associated with urticaria. It must be pointed out that the episodes of urticaria were not correlated to those of angioedema. This clinical finding is typical of IH-AAE [19], and reflects a real-life setting. Moreover, follow-up assessments based on telephone interviews carry a high risk of recall bias. In addition, this is a retrospective and monocentric study.

## 5. Conclusions

This study confirms that while IH-AAE is a benign and self-limiting condition, it can sometimes last for several years. Over time, the number of episodes per month decreases or, at most, remains unchanged. No patients reported disease worsening. The frequency of attacks at onset does not correlate with the possibility of recovery or the remission time.

More extensive studies could focus on diagnosing and managing IH-AAE in childhood to confirm these findings.

## Figures and Tables

**Table 1:** The characteristics of the population and the disease manifestations analyzed during follow-up.

Variable	Population Examined at Follow-Up (36)					
	No. (%)	Min	25th Pctl	Median	75th Pctl	Max
Age (y)	36	0.8	6.8	9.5	12.2	17.1
Ep/month	36	0.1	0.8	1.7	3.9	15
Male	22 (61%)					
Female	14 (39%)					
Urticaria						
yes	12 (33.3%)					
no	24 (66.7%)					
Antihistamine						
regularly	19 (52.8%)					
on demand	15 (41.7%)					
not indicated	2 (5.5%)					
Sites						
lips	25 (69.4%)					
eyelids	18 (50.0%)					
face	5 (13.9%)					
tongue/cheeks	5 (13.9%)					
hands/feet	3 (8.3%)					
genitals	2 (5.6%)					
limbs	2 (5.6%)					
back/gluteus	2 (5.6%)					
neck	2 (5.6%)					
gastrointestinal	0 (0.0%)					
larynx/airways	0 (0.0%)					
Recurrence						
low	14 (38.9%)					
intermediate	13 (36.1%)					
high	9 (25.0%)					

## Data Availability

Dataset available on request from the authors due to private reason.

## Abbreviations

The following abbreviations are used in this manuscript:

IH-AAE	Idiopathic Histaminergic Acquired Angioedema
C1-INH human	C1-esterase inhibitor
AE	Angioedema
